# Quality of life among patients with moderate to advanced chronic kidney disease in Ghana - a single centre study

**DOI:** 10.1186/s12882-019-1316-z

**Published:** 2019-04-08

**Authors:** Elliot K. Tannor, Betty R. Norman, Kwame K. Adusei, Fred S. Sarfo, Mogamat R. Davids, George Bedu-Addo

**Affiliations:** 1 Renal Unit, Department of Internal Medicine, Komfo Anokye Teaching Hospital, Kumasi, Ghana; 2 Department of Medicine, Kwame Nkrumah University of Science and Technology, Kumasi, School of Medical Sciences, Kumasi, Ghana; 3 Research and Development Unit, Komfo Anokye Teaching Hospital, Kumasi, Ghana; 4 Division of Nephrology, Department of Medicine, Stellenbosch University and Tygerberg Hospital, Cape Town, South Africa

**Keywords:** Quality of life, Chronic kidney disease, Ghana

## Abstract

**Background:**

The prevalence of chronic kidney disease (CKD) is increasing worldwide and in Africa. Health related quality of life (QOL) has become an essential outcome measure for patients with CKD and end stage renal disease (ESRD). There is growing interest worldwide in QOL of CKD patients but paucity of data in Ghana. This study sought to assess QOL in patients with moderate to advanced CKD (not on dialysis) and establish its determinants.

**Methods:**

We conducted a cross sectional observational study at the renal outpatient clinic at Komfo Anokye Teaching Hospital (KATH). We collected demographic, clinical and laboratory data. A pretested self-administered Research and Development corporation (RAND®) 36-Item Health Survey questionnaire was administered and QOL scores in physical component summary (PCS) and mental component summary (MCS) were computed. Determinants of QOL were established by simple and multiple linear regression. *P* value of < 0.05 was considered statistically significant.

**Results:**

The study included 202 patients with CKD not on dialysis. There were 118(58.5%) males. Mean age was 46.7 ± 16.2 years. The majority, 165(81.7%) of patients were on monthly salaries of less than GHS 500 (~USD 125). Chronic glomerulonephritis was the most common cause of CKD in 118 (58.5%) patients followed by diabetes mellitus in 40 (19.8%) patients and hypertension in 19 (9.4%) patients. The median serum creatinine was 634.2 μmol/L (IQR 333–1248) and the median eGFR was 7 ml/min/1.73m^2^ (IQR 3–16). The most common stage was CKD stage 5 accounting for 143 (71.1%), followed by CKD stage 4 with 45 (22.4%) of cases and 13 (6.5%) of CKD stage 3. The overall mean QOL score was 40.3 ± 15.4. MCS score was significantly lower than PCS score (37.3 ± 10.8 versus 43.3 ± 21.6, *P* < 0.001). Multiple linear regression showed that low monthly income (*p* = 0.002) and low haemoglobin levels (*p* = 0.003) were predictive of overall mean QOL.

**Conclusion:**

Patients with moderate to advanced CKD had low-income status, presented with advanced disease and had poor QOL. Anaemia and low-income status were significantly associated with poor QOL.

## Background

The prevalence of chronic kidney disease (CKD) is increasing worldwide and in Africa [1, 2]. The global prevalence is estimated to be 10–13% [1, 3]. The prevalence of CKD in sub-Saharan Africa was found to be 13.9% in a meta-analysis [4]. Chronic glomerulonephritis together with non-communicable diseases such as hypertension and diabetes mellitus account for the increasing prevalence of CKD in Sub-Saharan Africa and in Ghana [5]. CKD is associated with increasing morbidity and mortality and also known to impact negatively on quality of life (QOL) [6, 7].

QOL is defined by the World Health Organisation (WHO) as “an individual’s perception of their position in life in the context of the culture and value system where they live, and in relation to their goals, expectations, standards, and concerns” [8]. It is a comparison between patients’ expectations and reality. QOL also reflects treatment quality in patients with CKD and end stage renal disease (ESRD) since adapting to CKD involves physical, psychological and social processes to achieve the best outcomes [9]. QOL also represents a functional impact of a disease or its treatment on the subjective feeling of the affected individual about their physical, mental, spiritual, emotional, social and functional wellbeing [10]. The collection of QOL data helps patients understand their disease and the implications of treatment on their health [11].

Progression to ESRD also serves as an important burden on health care systems [12]. Management of ESRD involves haemodialysis (HD), peritoneal dialysis (PD), renal transplantation and conservative management. Conservative management is now established as a treatment option among geriatric patients in some developed countries [13].

In Ghana, like most countries in Sub-Saharan Africa, most patients with ESRD cannot afford renal replacement therapy (RRT). There is lack of governmental support for chronic dialysis and most patients have to pay out of pocket to survive. Ghana has a national health insurance scheme that covers some medical conditions but not chronic haemodialysis. In most cases, patients and their relatives may need to solicit for funds from their families, community, corporate organizations and religious groups to enable them go through with their haemodialysis sessions and plan renal transplantation outside the country as Ghana currently does not have a renal transplant programme. The social support system for patients with CKD is mainly by their families, community, work place and religious groups.

In majority of cases, conservative management is not the patient’s choice as seen in developed countries. When conservation management is resorted to in Ghana, medications are prescribed to make patients comfortable both on admission and after discharge from hospital. They are extensively counselled about end of life decisions. Palliative care is not free in Ghana. Patients and or their families have to pay for palliative care and treatments if medications or procedures performed are not covered by the national health insurance scheme. This worsened the financial burden on patients and in some situations, they just request to be discharged home against medical advice.

Haemodialysis, which is the most common form of RRT in Ghana, only provides a partial solution to the deteriorating kidney function. Patients still have to deal with some symptoms of the disease, many medications, a strict diet, regular hospital visits, multiple admissions and changes in body image when dialysis catheters are inserted or arteriovenous fistulae are made.

With many patients with ESRD unlikely to gain access to RRT in Ghana mainly because of financial constraints, it is imperative to ensure that CKD patients with advanced disease have the best QOL when being managed. There has been growing interest lately in improving the QOL of patients with kidney disease worldwide and not just on survival [14–16] but very few studies have focused on QOL of patients with CKD and ESRD in Ghana.

We therefore set out to establish the QOL of patients with moderate to advanced CKD in Ghana and determine the predictors of QOL. This will aid in the holistic management of patients with CKD and ESRD more especially in the absence of RRT. It will serve as the baseline data for further QOL studies in patients on RRT in Ghana.

## Methods

We conducted a cross sectional study at renal out-patient clinic of the Komfo Anokye Teaching Hospital (KATH). KATH is a 1200 bed capacity tertiary level health facility located in Kumasi in the Ashanti region of Ghana. KATH runs a twice-a-week out-patients’ clinic for patients with CKD and ESRD. Due to poor economic status, most patients seen are unable to afford RRT and are managed conservatively even in ESRD with indications for haemodialysis. Haemodialysis is the main RRT modality in Ghana. Ghana does not have a renal transplant program and does not offer peritoneal dialysis as a treatment modality for adult patients with ESRD. The study was conducted from October 2016 to August 2017.

Participants were included in the study if they had confirmed CKD with estimated glomerular filtration rate (eGFR) of less than 60 ml/min/1.73m^2^ as estimated by the Modification of Diet in Renal Disease (MDRD) equation [17] and gave a written informed consent. They were then staged according to Kidney Disease Outcomes Quality Initiative (KDOQI) [18] and Kidney Disease Improving Global Outcome (KDIGO) criteria [19]. Patients with acute kidney injury, on RRT or with concurrent chronic illness such as malignancy, chronic liver disease or stroke unrelated to the CKD or admitted to hospital within the last 1 month were excluded from the study. The clinical aetiology of CKD as diagnosed by the attending nephrologist was recorded from clinical notes.

A structured questionnaire was administered to each patient to obtain the following data: age, sex, level of education, marital status, occupation and monthly salary in Ghanaian Cedis (GHS). Clinical and laboratory data such as blood pressure, haemoglobin level, mean corpuscular volume (MCV), mean corpuscular haemoglobin (MCH), serum urea and serum creatinine were also recorded.

Anaemia was defined as haemoglobin level of less than 12 g/dl as suggested by the KDIGO guidelines [19]. Participants were then required to complete a pre-tested, validated, structured and a self-administered RAND 36-Item Health Survey (Version 1.0) [20]. The Short Form (SF-36) scoring system has been widely used and validated in most European countries. It was developed in the United States during the medical outcome survey between 1985 and 1992 and has been translated into 55 languages [21]. The questionnaire includes eight concepts: physical functioning, bodily pain, role limitations due to physical health problems, role limitations due to personal or emotional problems, emotional well-being, social functioning, energy/fatigue, and general health perceptions. It also includes a single item that provides an indication of perceived change in health. Scores range from 0 to 100 with 100 representing the best QOL score and zero representing the worst QOL scores.

Data was entered into a Microsoft software Excel® 2016 by a data entry clerk. The data was then cleaned and backed-up onto an external drive. Double entry was done to ensure the quality of the data obtained. Data collected was then exported into the statistical package Stata® version 13 [22]. Descriptive analysis was performed as means ± standard deviations if data is normally distributed and medians and inter-quartile ranges if data was not normally distributed. Percentages and frequencies were used to describe variables where appropriate.

Chi square test was used for comparisons between groups with categorical variables and an unpaired Student t test was used to compare groups with normally distributed continuous variables and Mann Whitney U test when not normally distributed. Simple and multiple linear regression analysis were then performed on the physical component summary (PCS) scores and the mental component summary (MCS) scores for determinants of QOL. Statistically significant variables identified in the simple linear regression were then included in a multiple linear regression model to establish the independent predictors for the QOL among patients with moderate to advanced CKD. Statistical significance was set at a two tailed *p*-value of less than 0.05.

Ethical approval from the Committee on Human Research, Publications and Ethics (CHRPE), Kwame Nkrumah University of Science and Technology and KATH was obtained before the commencement of the study.

## Results

The study included 202 patients with moderate to advanced CKD not on dialysis. There were 118 (58.4%) males. The mean age of participants was 46.7 ± 16.2 years. Ninety-six (47.5%) of patients were fully employed and 83 (41.1%) were unemployed. Majority, 176 (87.1%) did not attain tertiary level of education. The vast majority, 165 (81.7%) of patients were on monthly salaries of less than GHS 500 (~USD 120). Socio-demographic data of participants are shown in Table 1. The most common cause of CKD was chronic glomerulonephritis accounting for 118 (58.4%) of patients followed by diabetes mellitus in 40 (19.8%) patients and hypertension in 19 (9.4%) patients. The most common stage of presentation of CKD was stage 5 with eGFR of less than 15 ml/min/1.73m^2^ accounting for 143 (71.1%) of the cases. The median eGFR at presentation was 7 ml/min/1.73m^2^ (IQR 3–16). Most, 187(92.6%) of patients had hypertension. The mean haemoglobin level was 9.2 ± 2.5 g/dl. All as shown in Table 1.

**Table 1:** Characteristics of patients with moderate to advanced chronic kidney disease at Komfo Anokye Teaching Hospital *n* = 202

Characteristic	*n* = 202
Age (yrs) μ SD	46.7 ± 16.2
Male Gender n (%)	118 (58.4%)
Marital status	
Married n (%)	137 (67.8)
Single n (%)	42 (20.8)
Divorced/separated n (%)	12 (5.9)
Widowed n (%)	11(5.5)
Educational level	
No formal education n (%)	22 (10.9)
Primary education n (%)	21 (10.4)
Junior high school n (%)	56 (22.7)
Senior high school n (%)	77 (38.1)
Tertiary n (%)	26 (12.9)
Employment status	
Full time employment n (%)	96 (47.5)
Part time employment n (%)	4 (2.0)
Unemployed n (%)	83 (41.1)
In training n (%)	4 (2.0)
Retired n (%)	15 (7.4)
Monthly income status	
Less than GHS 500 n (%)	165 (81.7)
GHS 500–1499 n (%)	20 (9.9)
GHS 1500–2499 n (%)	9 (4.5)
GHS 2500–3499 n (%)	2 (1.0)
Greater than GHS 3500 n (%)	6 (3.0)
Cause of CKD	
Chronic glomerulonephritis n (%)	118 (58.4)
Diabetes mellitus n (%)	40 (19.8)
Hypertension n (%)	19 (9.4)
Polycystic kidney disease n (%)	14 (6.9)
HIVAN n (%)	5 (2.5)
Obstructive uropathy n (%)	2 (1.0)
Others n (%)	4 (2.0)
Stage of CKD	
Stage 3 n (%)	13 (6.5)
Stage 4 n (%)	45 (22.4)
Stage 5 n (%)	143 (71.1)
Systolic BP mmHg μ SD	152.3 ± 30.0
Diastolic BP mmHg μ SD	88.6 ± 19.8
Hypertension n (%)	187 (92.6)
Laboratory characteristics	
Haemoglobin levels (g/dL)) μ SD	9.2 ± 2.5
Anaemia (Hb < 12 g/dL) n (%)	164 (81.2)
MCV fL μ SD	81.4 ± 7.6
MCH pg/cell μ SD	28.0 ± 3.2
Serum Urea mmol/L M (IQR)	19.2 (12.5–30.5)
Serum Creatinine umol/L M (IQR))	634.2 (333–1248)
GFR ml/min/1.73m^2^ M (IQR)	7 (3–16)
Medications used	
ACEi/ARBs n (%)	141 (70.2)
Calcium channel blockers n (%)	161 (80.1)
Diuretics n (%)	160 (79.6)
Beta- blockers n (%)	56 (27.9)
Hydralazine n (%)	56 (27.9)
Methyldopa n (%)	80 (39.8)
ESA n (%)	19 (9.4)
Intravenous iron n (%)	4 (2.0)

*Yrs* years, *SD* standard deviation, *μ* mean, *n.* number, *GHS* Ghanaian cedis, *USD* United States dollars, *MCV* mean corpuscular volume, *MCH* mean corpuscular haemoglobin, *SD* standard deviation, *μ* mean, *IQR* interquartile range, *M* median, *BP* blood pressure, *GFR* glomerular filtration rate, *fL* femtolitres, *pg* pigogram, *CKD* chronic kidney disease, *HIVAN* human immunodeficiency virus associated nephropathy, *ACEi* angiotension converting enzyme inhibitor, *ARBs* angiotensin II receptor blocker, *ESA* erythropoietin stimulating agent, *others* chronic schistosomiasis and posttraumatic nephrectomy

The most commonly used antihypertensive class was calcium channel blockers used by 161 (80.1%) of patients and diuretics used by 160 (79.6%) of patients. Only 19 (9.4%) of patients with moderate to advanced CKD were on subcutaneous erythropoietin and 4 (2%) were on intravenous iron preparations. All as shown in Table 1.

The overall mean QOL score was 40.3 ± 15.4. Mean mental component summary (MCS) score (37.3 ± 10.8, 95% CI [35.8–38.8]) was significantly lower than the mean physical component summary (PCS) scores (43.3 ± 21.6, 95% CI [40.3–46.3], *P* < 0.001). The scores for the various sub-domain are shown in Table 2.

**Table 2:** Quality of life scores for physical component summary and mental component summary and their subdomains of patients with CKD *n* = 202

Principal domains	Sub-domain	Mean (SD)	Total mean (SD)
	Physical health	44.8 ± 30.0	
	Role physical	12.4 ± 33.0	
	Bodily pain	59.2 ± 29.0	
	General health	56.8 ± 15.8	
Mean physical component summary scores			43.3 ± 21.6
	Role emotional	12.4 ± 33.0	
	Social function	46.2 ± 20.7	
	Emotional wellbeing	40.7 ± 9.8	
	Energy	50.1 ± 9.1	
Mean Mental component summary score			37.3 ± 10.8
Overall mean Quality of life score			40.3 ± 15.4

Patients with low monthly income less than GHS 500 (~USD 125) had a significantly lower overall mean QOL score than those with higher monthly income above GHS 500 (38.5 ± 14.1 versus 48.4 ± 18.3, *p* < 0.001). Patients with anaemia also had a significantly lower overall mean QOL score as compared to those with haemoglobin levels greater than 12 g/dl (38.3 ± 13.6 versus 49.2 ± 19.3, p < 0.001). Patients with low mean corpuscular volume (MCV) had a significantly lower overall mean QOL score 36.9 ± 10.7 as compared to those with normal or high MCV (42.8 ± 17.7, *p* = 0.007). Patients with CKD stage 5 also had a significantly lower overall mean QOL score as compared to those presenting with CKD stages 3 and 4 (37.9 ± 13.6 versus 46.4 ± 17.9, *p* < 0.001). Patients with serum urea greater than 20 mmol/L as compared to those with urea less than 20 mmol/L also had significantly lower overall mean QOL scores (36.1 ± 13.1 versus 44.0 ± 16.7, *p* = 0.004). All as shown in Table 3.

**Table 3:** Independent variables and their association with the overall mean quality of life among patients with moderate to advanced chronic kidney disease *n* = 202

Variable	Mean ± SD	Frequency	*P* value
Age (yrs) ≥50	39.8 ± 15.5	93	0.687
Age (yrs) < 50	40.7 ± 15.4	109	
Female gender	38.9 ± 14.2	84	0.268
Male gender	41.3 ± 16.2	118	
Above JHS	41.1 ± 16.2	103	0.486
JHS and below	39.5 ± 14.5	99	
Employed	39.9 ± 15.9	100	0.673
Unemployed	40.8 ± 15.0	102	
Monthly income(GHS) < 500	38.5 ± 14.1	165	< 0.001
Monthly income(GHS) ≥ 500	48.4 ± 18.3	37	
Married	41.0 ± 15.6	137	0.373
Unmarried	38.9 ± 15.0	65	
Haemoglobin < 12 g/dL	38.3 ± 13.6	164	< 0.001
Haemoglobin level ≥ 12 g/dL	49.2 ± 19.3	38	
MCV < 80 fL	36.9 ± 10.7	85	0.007
MCV ≥ 80 fL	42.8 ± 17.7	117	
MCH < 27 pg/cell	39.9 ± 14.4	62	0.783
MCH ≥ 27 pg/cell	40.5 ± 15.9	140	
CKD stage 5	37.9 ± 13.6	143	< 0.001
CKD Stage 3 and 4	46.4 ± 17.9	58	
Serum urea≥20 mmol/L	36.1 ± 13.1	90	0.004
Serum urea < 20 mmol/L	44.0 ± 16.7	101	
ESA use	41.9 ± 12.9	19	0.651
Not on ESA	40.2 ± 15.7	182	
IV iron use	50.4 ± 19.6	4	0.191
No IV iron	40.2 ± 15.3	197	

*JHS* junior high school, *MCV* mean corpuscular volume, *MCH* mean corpuscular haemoglobin, *GHS* Ghana Cedis, *CKD* chronic kidney disease, *ESA* erythropoietin stimulating agent, *IV* intravenous, *SD* standard deviation

### Linear regression analysis

In simple linear regression, PCS scores were associated with low monthly income status (*p* < 0.001), low haemoglobin (p < 0.001), low MCV (0.019), serum urea > 20 mmol/L (p < 0.001) and CKD stage 5 (p < 0.001). All as shown in Table 4.

**Table 4:** Simple linear regression analysis of physical component summary scores in patients with moderate to advanced chronic kidney disease *n* = 202

Variable	Coefficient	Standard error	t	95% CI	*P* value
Age	−0.02	0.09	−0.25	− 0.21–0.16	0.806
Educational level	1.61	3.04	0.53	−4.38–7.60	0.597
Employment status	−1.23	3.04	−0.40	−7.22–4.77	0.687
Low income	−13.61	3.81	−3.57	−21.13–−6.09	< 0.001
Marital status	3.49	3.24	1.07	−2.91–9.90	0.284
Hypertension	−5.35	3.28	−1.63	−11.81–1.11	0.104
Hemoglobin	2.97	0.57	5.23	1.85–4.09	< 0.001
MCV	0.46	0.19	2.36	0.07–0.85	0.019
MCH	0.67	0.48	1.40	− 0.27–1.61	0.163
Uraemia	−11.87	3.04	−3.89	−17.89–−5.86	< 0.001
CKD stage 5	−12.63	3.25	−3.88	−19.04–−6.22	< 0.001
ESA use	3.03	5.21	0.58	−7.24–13.30	0.561
IV Iron use	6.25	10.91	0.57	−15.27–27.77	0.567

*MCV* mean corpuscular volume, *MCH* mean corpuscular haemoglobin, *CKD 5* chronic kidney disease with GFR < 15 ml/min/1.73m^2^, *ESA* erythropoietin stimulating agent, *IV* intravenous, *CI* confidence interval, *uraemia* serum urea > 20 mmol/L

Simple linear regression analysis of mental component summary scores were associated with low monthly income status (*p* = 0.002), CKD stage 5 (*p* = 0.009), low haemoglobin levels (p < 0.001), low MCV (*p* = 0.037) serum urea > 20 mmol/L (*p* = 0.015) and the use of IV iron preparation (*p* = 0.009) as shown in Table 5.

**Table 5:** Simple linear regression analysis of mental component summary scores in patients with moderate and advanced chronic kidney disease *n* = 202

Variable	Coefficient	Standard error	t	95% confidence interval	*P* value
Age	−0.01	0.05	−0.01	− 0.93–0.09	0.995
Educational level	1.42	1.51	0.94	− 1.56–4.41	0.348
Employment status	−0.61	1.51	−0.40	−3.60–2.38	0.690
Low income	−6.08	1.91	−3.18	−9.84–−2.30	0.002
Marital status	0.66	1.62	0.41	−2.54–3.86	0.685
Hypertension	−1.70	1.64	−1.03	−4.93–1.54	0.303
Hemoglobin	1.15	0.29	3.95	0.57–1.72	< 0.001
MCV	0.21	0.10	2.10	0.01–0.40	0.037
MCH	0.32	0.24	1.35	−0.15–0.80	0.178
Uraemia	−3.78	1.55	−2.45	−6.83–−0.73	0.015
CKD stage 5	−4.36	1.65	−2.64	−7.62–1.10	0.009
ESA use	0.34	2.60	.0.13	−4.76–5.48	0.895
IV Iron	14.12	5.35	2.64	3.56–24.69	0.009

*MCV* mean corpuscular volume, *MCH* mean corpuscular haemoglobin, *CKD 5* chronic kidney disease with GFR < 15 ml/min/1.73m^2^, *ESA* erythropoietin stimulating agent, *IV* intravenous, *CI* confidence interval, *uraemia* serum urea > 20 mmol/L

Simple linear regression of the overall QOL scores showed low haemoglobin level (*P* < 0.001), low MCV (0.0179), uraemia (p < 0.001), low monthly income status (*p* = 0.004) and CKD stage 5 (*P* = 0.003) as significant determinants as shown in Table 6.

**Table 6:** Simple linear regression analysis of independent variables of overall mean quality of life score in patients with chronic kidney disease *n* = 202

Variable	Coefficient	Standard error	t	95% confidence interval	*P* value
Age	− 0.01	0.07	0.86	−0.14–−0.12	0.862
Educational level	1.52	2.17	0.70	−2.76–5.80	0.486
Employment status	−0.92	2.17	−0.42	−5.20–3.37	0.673
Low income	−0.84	2.72	−3.62	−15.21–4.48	< 0.001
Marital status	2.07	2.32	0.89	−2.50–6.65	0.373
Hypertension	−3.52	2.34	−1.50	−8.14–1.10	0.134
Hemoglobin	2.06	0.41	5.06	1.26–2.86	< 0.001
MCV	0.34	0.14	2.39	0.06–0.62	0.018
MCH	0.50	0.34	1.45	−0.18–1.17	0.148
Uraemia	−7.83	2.19	−3.58	−12.15–−3.51	< 0.001
CKD stage 5	−8.50	2.33	−3.64	−13.10–−3.90	< 0.001
ESA use	1.69	3.72	0.45	−5.65–9.03	0.650
IV Iron	10.19	7.77	1.31	−5.13–25.51	0.191

*MCV* mean corpuscular volume, *MCH* mean corpuscular haemoglobin, *CKD 5* chronic kidney disease with GFR < 15 ml/min/1.73 m2, *ESA* erythropoietin stimulating agent, *IV* intravenous, *uraemia* urea> 20 mmol/L

### Multiple linear regression analysis

Multiple linear regression analysis showed that low monthly income status (*p* = 0.002) and haemoglobin levels were significant predictors of mean PCS scores. Multiple linear regression revealed the predictors of mean MCS scores as low monthly income status (*p* = 0.004), low haemoglobin (*p* = 0.025) and the use of intravenous iron preparation (*p* = 0.001). Predictors of overall mean QOL score were low-income status (*p* = 0.002) and low haemoglobin level (*p* = 0.003) in multiple linear regression all as shown in Table 7.

**Table 7:** Multiple linear regression analysis of independent variables for physical, mental component summary and overall QOL scores in patients with CKD

Variable	Beta coeff	Standard error	t	95% CI	*P* value
Physical	component	Summary	score	Adjusted R^2^ =	0.18
Low income	−0.21	3.52	−3.18	−19.69–−4.62	0.002
CKD stage 5	−0.07	3.97	−0.93	−11.53–4.13	0.352
haemoglobin	0.24	0.67	3.13	0.77–3.4	0.002
MCV	0.77	0.20	1.12	−0.17–0.61	0.264
uraemia	−0.13	3.33	−1.73	−12.31–0.81	0.209
Mental	Component	summary	Score	Adjusted R^2^ =	0.15
Low income	−0.20	1.94	−2.91	−9.47–−1.81	0.004
CKD stage 5	−0.04	2.02	−0.41	−4.82–3.14	0.689
haemoglobin	0.18	0.34	2.26	−0.10–1.43	0.025
MCV	0.13	0.10	1.82	−0.02–0.38	0.070
uraemia	−0.09	1.70	−1.13	−5.27–1.44	0.261
IV iron use	0.23	5.13	3.30	6.81–27.04	0.001
Overall	QOL	Score		Adjusted R^2^ =	0.17
Low income	−0.21	2.75	−3.16	−14.11–−3.27	0.002
CKD stage 5	−0.07	2.86	−0.85	−8.05–3.22	0.398
haemoglobin	0.24	0.48	3.02	0.50–2.40	0.003
MCV	0.09	0.14	1.27	−0.10–0.46	0.205
uraemia	−0.11	2.39	−1.49	−8.28–1.16	0.138

*MCV* mean corpuscular volume, *CKD 5* chronic kidney disease with GFR < 15 ml/min/1.73 m2, *IV* intravenous, *SD* standard deviation, *QOL* quality of life, *CI* confident interval, *uraemia* serum urea> 20 mmol/L, *coeff* coefficient

## Discussion

To our knowledge, this is the first ever study to assess the quality of life among patients with moderate to advanced CKD not on dialysis using the RAND® 36-Item Health Survey questionnaire in Ghana. Our study showed that the QOL of patients with moderate to advanced CKD was poor. Low-income status, anaemia and CKD stage 5 were identified to be significantly associated with poor QOL.

There was a slight predominance of males over females as shown in other studies [23, 24]. Male gender has been associated with faster progression of renal disease as compared to females [25]. Worse progression of CKD in males have also been validated in a meta-analysis of eight studies involving 2229 patients [24]. The mean age in our cohort was 46 years similar to an earlier study in Ghana [23] but lower than shown in the meta-analysis with a mean age of 48.1 years [24]. The mean age of CKD patients in developed countries has been shown however to be higher than 50 years in most studies [26–28].

The overall mean QOL score in patients with moderate to advanced CKD not on dialysis in our cohort was lower as compared to a study by Perlman et al. [29]. This may be due to low median eGFR of 7 ml/min/1.73m^2^ in our patients as compared to 23 ml/min/1.73m^2^ in their study. The QOL scores were low for both physical component summary (PCS) and mental component summary (MCS) scores. MCS scores were significantly lower than PCS (37.3 versus 43.3; *p* < 0.001). This may be due to the greater impact of the disease burden on the mental status of patients that may be overlooked by attending physicians and nephrologists in most cases. Management of patients with CKD by their physicians mainly focus on making patients physically healthy with less emphasis on the mental and psychological impact of the disease on their general wellbeing. Focus on the mental health of patients with CKD may therefore be necessary to improve their QOL. It has been shown that the psychological impact of chronic diseases including CKD is underestimated by health care workers and patients relatives [11].

Low-income status was identified as a predictor of poor QOL as also shown in other studies [30, 31]. Most patients with CKD were of low monthly income status about or just minimally higher the minimum wage currently in Ghana. Over 80% of patients were on a monthly income of less than GHS 500 (USD 125). The minimum wage in Ghana is currently pegged at GHS 8.8 (∼USD 2.2) per day with a monthly income of about USD 66 [32]. Furthermore, majority (87.1%) of patient’s did not get to tertiary level academically. These patients may therefore lack the requisite knowledge in the prevention and early management of CKD. Patients with CKD also have poor health seeking behaviour due to their low-income status and low literacy rate. They therefore present late with very advanced CKD as shown in our study. Patients with CKD are also unable to afford the cost of management of CKD and RRT when they progress to ESRD due to their low socioeconomic status [33]. The cost of haemodialysis in Ghana is currently between GHS190 to 260 (~USD 50–65) per session. The cost of chronic haemodialysis is not covered by the national health insurance scheme (NHIS). Practically, the NHIS only pays for acute haemodialysis in very few situations (if any), up to GHS 850 (∼USD 265) [33] irrespective of the number of sessions required for recovery. Patients are also required to buy their medications such as anti-hypertensives, haematinics, intravenous iron preparations and subcutaneous erythropoietin stimulation agents (ESA) and carry out routine laboratory investigations to assess their response to treatment. Patients’ inability to afford treatment especially for anaemia may negatively affect their QOL as shown in our study.

Prevalence of anaemia among our patients with moderate to advanced CKD was 81.2% as shown in other studies [23, 34]. Anaemia is a common presentation in patients with CKD and progressively worsens with advancing renal disease. Most patients had normocytic normochromic anemia which has been described as the characteristic form of anaemia in renal disease due to decreased production of erythropoietin since 1950 [35]. Lately, increase in hepcidin as a result of chronic inflammation has been described in the pathogenesis of anaemia in patients with CKD [36]. Forty-two percent of patients presented however with low MCV anaemia which may have resulted from anorexia [37], occult gastrointestinal bleeding, iron deficiency or the presence of hepcidin causing anaemia of chronic disease [36]. Anaemia could also be due to low red cell survival which has also been reported in patients with CKD [37]. Iron absorption is poor in patients with CKD making intravenous iron more beneficial in improving iron status in patients with kidney disease but most patients with moderate to advanced CKD cannot afford intravenous iron preparations. Anaemia could also be due to chronic bleeding from uraemia associated platelet dysfunction and frequent phlebotomy [37].

Unfortunately, only 9% of the patients in our cohort were on ESA. This is because most of the patients are of low socioeconomic status and cannot afford the ESA. ESA is therefore less prescribed by attending physicians and nephrologists. ESAs have been used over the years with many benefits in improving symptoms, avoiding frequent haemotransfusions and to prevent complications associated with transfusions like iron overload, infections and transfusion related reactions [38–40]. ESA has been shown to be associated with hypertension, increased thrombosis and malignancies [41]. ESA use with haemoglobin target levels beyond 13 g/dL has not been shown to be beneficial and even associated with poorer outcomes in major studies [19, 39]. Anorexia in patients with CKD and vomiting associated with uraemia also leads to decreased intake and absorption of iron from the gut. Intravenous iron is warranted in some cases but the use of intravenous iron was also low due to its high cost and under-prescription by attending physicians. Only 4% patients in our cohort were on intravenous iron preparations.

Patients with haemoglobin levels less than 12 g/dl had poorer QOL as compared to patients without anaemia (38.3 ± 13.6 versus 49.2 ± 19.3; *p* < 0.001). Anemia has been shown in several studies to be associated with adverse clinical outcome [34] and affects QOL in patients with CKD [42, 43]. Anaemia was found to predict poor PCS scores, MCS scores and the overall mean QOL scores in multiple linear regression analysis as also shown in other studies in both developing and developed countries [44–46].

Low MCV was significantly associated with mean MCS scores (*p* = 0.037), PCS scores (0.019) and overall mean QOL (*p* = 0.018). This may be related to iron deficiency, which may have resulted from anorexia, occult blood loss from uraemic gastritis or bleeding diathesis from dysfunctional platelets seen in patients with CKD [37]. Low MCV was however insignificant in a multiple linear regression analysis for PCS, MCS scores and overall mean QOL scores and not a predictor of poor QOL.

The use of IV iron preparation was found to be significantly associated with MCS scores but not overall mean QOL or PCS scores. This may be because those who could afford IV iron were mainly of high-income status, which may have positively affected their MCS scores. The number of patients on intravenous iron preparations was however very small.

Majority (71%) of patients had CKD stage 5 as also shown in some studies in Africa [23, 47]. Patients reporting late with CKD stage 5 also had a poorer QOL as compared to those reporting with CKD stages 3 and 4. This may be because patients with CKD stage 5 present with more symptoms, greater disease burden and had significantly lower haemoglobin levels (8.4 versus 11.1, *P* < 0.001). Symptoms of uraemia such as anorexia, nausea and vomiting may have also contributed to their poor QOL.

QOL was however not associated with age, gender, educational status, employment status, marital status and mean corpuscular haemoglobin (MCH) in our cohort similar to that found by Suet et al. [48] who found no association with age and marital status. This differed from another study that found an association with age, gender and race [49]. Male gender, Asian ethnicity and poor nutritional status were found to be associated with poor QOL in another study [50]. Czyzewski et al. showed that increasing age, increased body mass index and unemployment were associated with lower QOL scores [51]. Patients with higher socioeconomic status, male gender and unmarried were shown by Alveres et al. in a large multi center study of over 3000 participants in Brazil to be associated with improved quality of life scores [31].

Our study had a number of limitations. Iron studies were not assessed due to financial constraints. Iron studies would have helped identify clearly if iron deficiency was the cause of the low MCV. The RAND® 36-Item questionnaire [20] was interpreted to few participants who could not read nor write. This may have introduced interviewer bias because the tool was supposed to be a self-administered questionnaire. It was also a tertiary hospital based study were the worst of cases are referred to very late. This may have led to selection bias in their poorer outcomes.

## Conclusions

The overall QOL in patients with moderate to advanced CKD was poor. MCS score was significantly poorer than PCS scores. Determinants of poor QOL were low economic status, patients with CKD stage 5 and anaemia. There is the need for governmental support for patients with CKD to improve their QOL. QOL assessment should be introduced for routine clinical review to identify those with poor QOL for proper management based on the determinants of poor QOL. A psychologist and or psychiatrists should be involved in the management of patients with moderate to advanced CKD to focus on mental components that affects the QOL to improve the overall health of patients with moderate to advanced CKD in Ghana.
